# Water and soil loss from landslide deposits as a function of gravel content in the Wenchuan earthquake area, China, revealed by artificial rainfall simulations

**DOI:** 10.1371/journal.pone.0196657

**Published:** 2018-05-03

**Authors:** Fengling Gan, Binghui He, Tao Wang

**Affiliations:** 1 College of Resources and Environment/Key Laboratory of Eco-environment in Three Gorges Region (Ministry of Education), Southwest University, Chongqing, China; 2 Central Southern China Electric Power Design Institute limited liability company of China Power Engineering Consulting Group, Wuhan, China; Durham University, UNITED KINGDOM

## Abstract

A large number of landslides were triggered by the Mw7.9 Wenchuan earthquake which occurred on 12th May 2008. Landslides impacted extensive areas along the Mingjiang River and its tributaries. In the landslide deposits, soil and gravel fragments generally co-exist and their proportions may influence the hydrological and erosion processes on the steep slopes of the deposit surface. Understanding the effects of the mixtures of soil and gravels in landslide deposits on erosion processes is relevant for ecological reconstruction and water and soil conservation in Wenchuan earthquake area. Based on field surveys, indoor artificial rainfall simulation experiments with three rainfall intensities (1.0, 1.5 and 2.0 mm·min^-1^) and three proportions of gravel (50%, 66.7% and 80%) were conducted to measure how the proportion of gravel affected soil erosion and sediment yield in landslide sediments and deposits. Where the proportion of gravel was 80%, no surface runoff was produced during the 90 minute experiment under all rainfall intensities. For the 66.7% proportion, no runoff was generated at the lowest rainfall intensity (1.0 mm·min^-1^). As a result of these interactions, the average sediment yield ranked as 50> 66.6> 80% with different proportions of gravel. In addition, there was a positive correlation between runoff generation and sediment yield, and the sediment yield lagging the runoff generation. Together, the results demonstrate an important role of gravel in moderating the mobilization of landslide sediment produced by large earthquakes, and could lay the foundation for erosion models which provide scientific guidance for the control of landslide sediment in the Wenchuan earthquake zone, China.

## 1. Introduction

The May 12, 2008, Wenchuan earthquake with a moment magnitude of Mw7.9 is one of the most devastating earthquakes in China. Ground shaking during the earthquake triggered a large number of landslides which induced soil erosion on the mountain sides, and produced loose debris in landslide deposits. As the result of this, the earthquake impacted the ecological system on the valley sides and within rivers, and threatened the production and livelihood security of the localities there. It is estimated that there were more than 60,000 landslide accumulation bodies [1]. Most of these landslide accumulation bodies were aggregate mixtures of rock and soil, which show a loose structure and low vegetation coverage and they were widely distributed in the upper reaches of the Minjiang River [2]. Landslide accumulation bodies are prone to secondary failure, which can destroy towns, roads, irrigation and other infrastructure and incur heavy damage to the life and property of local residents [3]. Under the condition of rainfall, runoff, earthquakes and other external forces, the landslide accumulation body is vulnerable to soil erosion, which not only causes a contribution of sediments into the river but also reduces the quality of the water of the Minjiang River. Conducting research on landslide accumulation bodies is of great significance for reconstruction in an earthquake area [4]. In recent years, many researchers were keen to determine their distribution, soil erosion and area of landslide deposits by earthquakes using the integration of Geographic Information Science (GIS) and Remote Sensing (RS) [5], and also from river sampling of the Minjiang and other major rivers and tributaries[6]. Besides, the widespread destruction of vegetation is also an important factor affecting the water and soil loss in earthquake area. The Environment for Visualizing Images (ENVI) and GIS software are mostly used to interpret the Thematic Mapper (TM) image data and satellite imagery from Google Earth for manually interpreting the landslide deposits [7]. After the Wenchuan earthquake, the Chinese National Bureau of Statistics released preliminary statistics which showed that soil erosion had occurred on 149.200 square kilometers of land which increased nearly 11.03% of its previous area. Earthquake-triggered landside deposits are mainly researched from a large scale perspective [8] and research on the processes and rates of soil erosion on the surface of landside deposits are somewhat lacking.

Because of their loose material, steep gradient and bare surface, earthquake induced landslides deposits can become major sources of sediment to rivers downstream during rainfall and runoff events [9–10]. Large rock fragments with particle sizes ranging from a few meters to several tens of meters generally exist in landslide deposits and are not very mobile. In contrast, smaller rock fragment particles ranging from millimeters to several centimeters in size are more easily removed by runoff. Based on the observations from Wenchuan earthquake area, the rock particle size changed significantly in the landslide deposits: the dominant grain sizes in the upper slope were fine sands, mid-gravel generally appeared in the mid-slope, and the large rock particle size existed primarily in the lower slopes [11–12]. The rock particle size of the soil-rock-mixture was highly variable, which led to a soil infiltration ability that was either promoted (a large rock particle size from a few meters to dozens of meters) or reduced (small rock particles from millimeters to several centimeters) [13]. But the rock particle size was not the most significant factor affecting the water and soil loss [14].

The structure and physical properties of a soil-rock-mixture differ from those of rock and soil, which are vulnerable to erosion and can trigger serious water and soil loss under the action of rainfall. In recent years, researchers have focused primarily on the characteristics of rock fragments, such as their position, shape, and size, which play important roles in runoff sediment yielding characteristics on the soil-rock-mixture with simulated rainfall or in scouring experiments [15]. There are indications that the soil erosion process is affected both directly and indirectly by rock fragments. Rock fragments can protect the surface soil from splashing and scattering by raindrops and intercept splashed sediment, which directly influences the soil erosion process [16]. Some studies have discovered the rock fragment characteristics such as geometry, size, and position impact the soil erosion in the soil-rock-mixture [17–18]. They found that the percentage of rock fragment cover is the most important factor that has a negative exponential relationship with runoff and sediment yield. However, the indirect effects of rock fragments can be very important for soil erosion. These effects include the multiplying effects of rock fragments on soil degradation (soil crust), soil physical properties, and hydrological process.

There are several studies which have sought to obtain a more comprehensive understanding of the impact of rock fragments on the soil erosion process under rainfall. Poesen et al. (1992) [19] found that the position and cover of rock fragments on the soil surface had a significant impact on soil runoff and sediment yield. Their results showed that the rock fragments increase runoff and sediment yield when well embedded in a surface seal (i.e. a top layer with essentially textural pore spaces). A negative relation occurs either where rock fragments are partly embedded in a top layer with structural porosity, or where the rock fragments rest on the surface of a soil having either textural or structural pore spaces. Meier and Hauer (2010) [20] indicated that the slope gradient affected both the rock fragment coverage and soil erosion. The sediment yield had a tendency for exponential decay with the gravel coverage and increased when the slope was gentle (3.5%). However, the relationship between sediment yield and gravel coverage was non-monotonic when the slope was steep (28.7%). In addition, previous research has shown that rock fragments can increase the quantity and volume of macrospores and promote the permeation of rainwater into the soil depth, which can maintain the water holding capacity [21]. On the one hand, the rock fragments had a positive impact on water infiltration capacity by increasing the soil porosity. On the other hand, the movement of soil and water was restricted by the rock fragment, which can increase the tortuosity of the medium and reduce the water flow. Most studies [22–23] were conducted on the effects of rock fragments, but few researches focused on a soil containing rock fragments which is relevant for landslide deposits.

In this paper, we studied landslide accumulation bodies from the perspective of soil erosion in the Wenchuan earthquake area and assessed the influence of rock fragments on soil erosion processes. The research objectives of this paper were as follows: (1) to quantify how the level of the weight proportion of gravel affected the runoff generation and sediment yield under the different rainfall intensities, (2) to examine the relationship between cumulative runoff and cumulative sediment with different rainfall intensity, and (3) to qualify the average runoff and sediment with different rainfall intensities and gravel proportions. Therefore, this paper provided a reasonable basis for evaluating earthquake landslide accumulation body as a soil erosion hazards, and for carrying out this study ecological restoration and earthquake disaster zone governance laid a theoretical foundation.

## 2. Materials and methods

### 2.1. Investigation area

**Fig 1:**
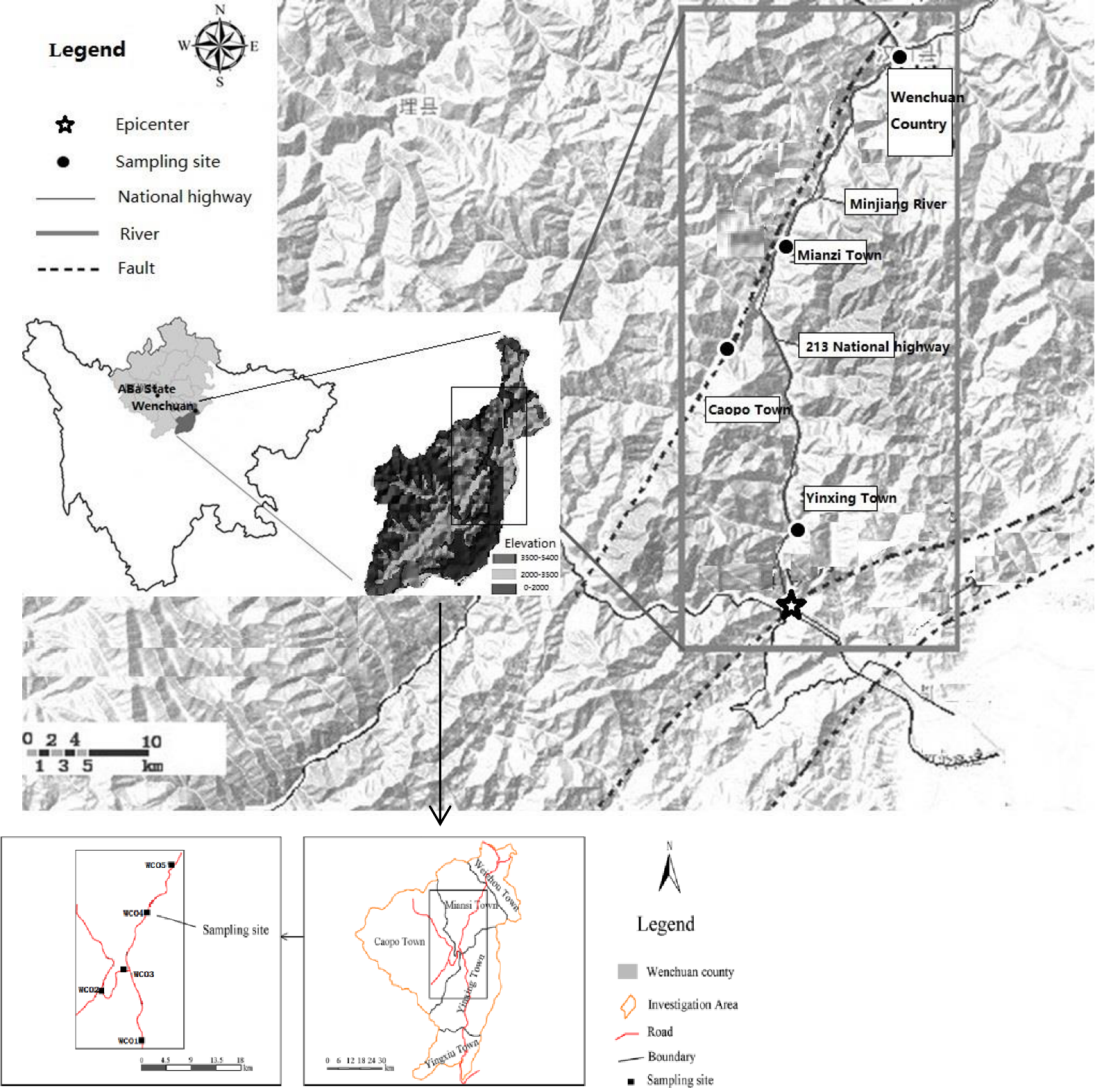
Location of study area and landslide sediment sample points.

The study is carried out on private land of each location (Fig 1), with the permissions from the land owners. All of the land uses are either for tillage or for landscaping with economic trees, and no specific permissions were required. According to the field investigation, the field does not involve with endangered or protected species.

The area [24] was conducted at the Mingjiang river (102°51′-103°44′E, 30°45′-31°43′N), Chengdu Plain, Sichuan Province, China (Fig 1). The study area is an important ecological barrier of the upper Yangtze River and the Wenchuan earthquake area, which is located in the upriver and headstream of the Mingjiang River, Fujiang River and Jianglingjiang River which provides significant ecosystem services for water conservation or soil and water conservation. The study area provides the water service safety for large population of the Chengdu Plain and the Sichuan Basin. The total area of the Caopo River is 528 km^2^ and it has a length of 45.5 km. The experimental area is located in the South Temperate Zone which has a typical semi-humid monsoon climate. The mean annual precipitation is 535 mm and the mean annual rainfall period is 149.6 days. The annual average relative humidity in this region is 69% and the mean annual wind speed is 2.8 m/s. The average frost-free period is 40.6 days. This region was once a landscape consisting primarily of trees; however, during the earthquake disaster, the primary vegetation was damaged, which led to poor soil structure characterized by low organic matter. The parent rock material is granite and the dominant crops are *wormwood*,*coriaria*, and *KoelreuteriabipinnataFranch*. The vegetation coverage rates are 0%~50% after the earthquake. Belonging to the landforms of highland and ravine, the general spatial pattern of the region is characterized by the high terrain in the northwest and the low terrain of the southeast, and the difference in altitude is more than 2000m. The landslide slopes are mainly 69.4%-86.1%, and the gravel particle size on the landslide accumulation experiences major changes with large bare rocks. The mass proportion of rock fragments is 55.43%-100% and the average proportion is 83.84%. The bulk density of the soil-rock-mixture is 1.43–1.80 g/cm^3^.

### 2.2 Soil sampling and processing

**Fig 2:**
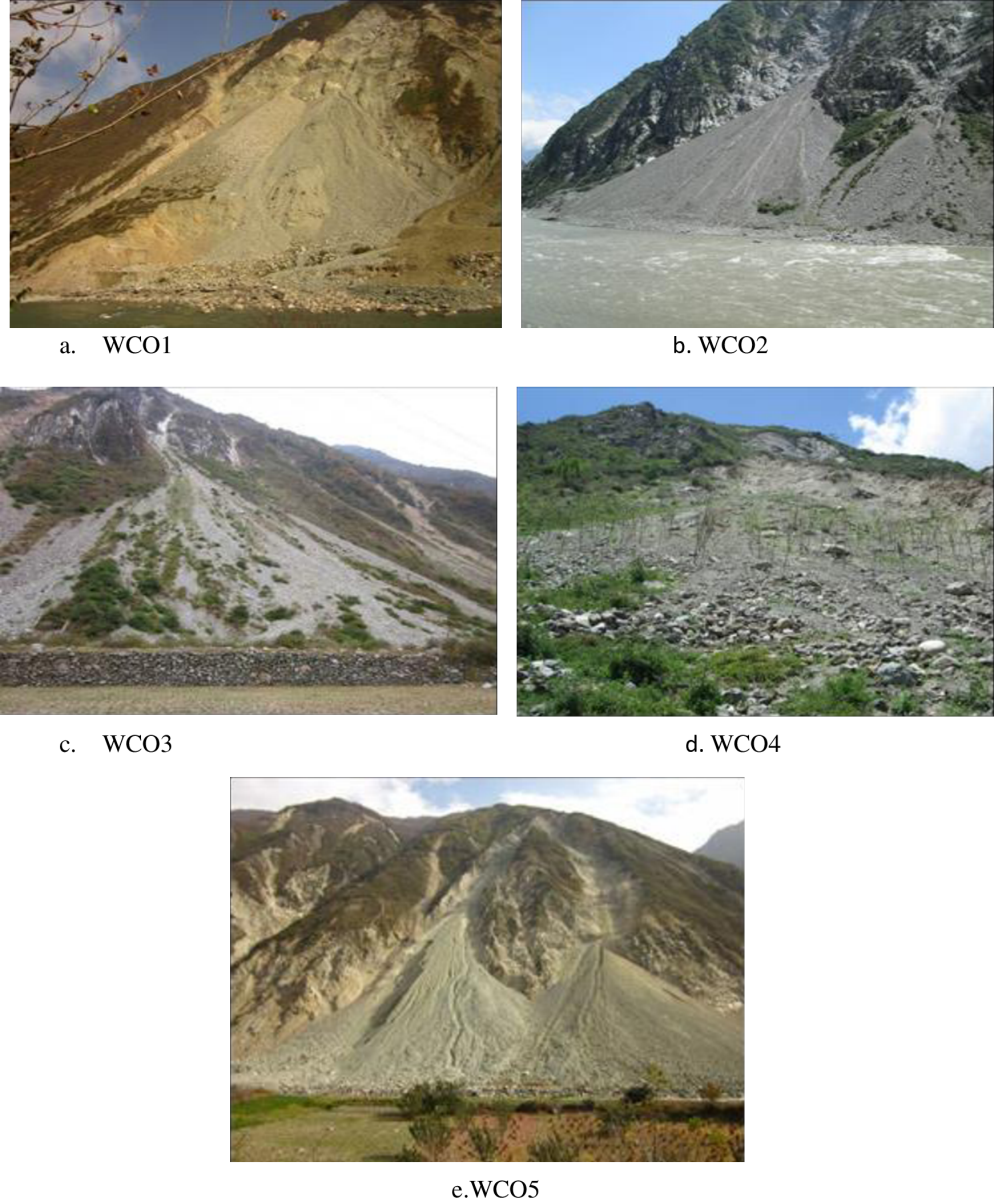
Field photograph of landslide accumulation.

The landslide sediment samples were collected and measured at 5 groups of typical landslide accumulation deposits (WCO1, WCO2, WCO3, WCO4, and WCO5) with similar physicochemical properties and topographical conditions in November 2013 and May 2014 (Fig 2). The samples were collected from the vertical positions of the slope at every 2m. The 0–40 cm layer was divided into four 10-cm-thick soil layers. Each landslide sediment samples in each soil layer was homogenized by hand mixing to create three duplicates. After recording the site properties, including the altitude, slope gradient, latitude, vegetation coverage and species, 1- kg soil samples were obtained by quartering, and transported to the laboratory, air-dried for 24h at 105°C and sieved using a 2mm screen by hand to remove stones, debris, plant roots and large animals such as worms. Meanwhile, an approximately 4 tons of soil sample were also collected for the rainfall simulator experiment which represented all the (WCO1) 5 typical landslide accumulations (Table 1).

**Table 1:** Basic information on the sampling points.

Labels	Geographic position	Elevation/m	Slope /(%)	Vegetation /%	Sampling methods	Weight percentage of gravel (%) (2-60mm)	Weight percentage of gravel (%) (2-60mm)
**WC01**	103°29′26″E、31°20′48″N	1199	81.1	0	4 layers	68.50	31.50
**WC02**	103°27′36″E、 31°17′21″N	1152	-	-	Surface layer	51.36	48.64
**WC03**	103°25′47″E、 31°15′15″N	1382	78.9	20~50	2 layers	63.41	36.59
**WC04**	103°29′26″E、 31°20′48″N	1199	69.4	0	2 layers	58.13	41.87
**WC05**	103°29′28″E、31°21′50″N	1237	86.1	0~20	2 layers	78.32	21.68

“-”presented the missing values due to lack of samples

Following Poesen et al (1991) [25] the soils containing rock fragments were divided into six categories: fine gravel (2–5 mm), middle gravel (5–20 mm), coarse gravel (20–76 mm), pebble (76–250 mm), rock (250–600 mm), and stone (>600 mm). Therefore, we classified the different particle diameters into four categories: coarse gravel (>60 mm), middle gravel (60–10 mm), fine gravel (10–2 mm), and sand (<2 mm). The air-dried soil samples were hand-sieved through 40–20 mm, 20–10 mm, 10–7 mm, 7–5 mm, 5–2 mm, and <2 mm sieves. We selected 60–10 mm particle size as the middle gravel and 10–2 mm particle size as the fine gravel. According to Poesen et al (1994) [26] the main proportion of the categories were mixed to form gravel. The rock-fragment part was mixed with the middle gravel and fine gravel by the proportion of 1:1. Then, the gravels and the soil (<2 mm) were completely mixed again. The bulk density of the soil-gravel mixture was approximately1.77 g·cm^-3^. The mass fraction of the initial water content was 8.25%-12.60%; thus the structure of the landslide accumulation was roughly the same as for the soil structure in the experiment.

### 2.3 Experimental setup and rainfall experiment

According to the practical measurement of the landslide accumulation in the Wenchuan earthquake area, the mountain slopes were mostly concentrated at 80.0%. Based on field investigation, we can see the weight percentages of soil in which diameter is less than 2 mm were 21.68–48.64%, while the weight percentages of gravels in which diameters are between 2 and 40 mm were 51.36–78.32% (Table 1). Therefore, the slope of the experiment was fixed at 80.6%, and the weight proportion of the gravel was set as 50%, 66.7% and 80%. The rainfall intensities were fixed at 1.0, 1.5, and 2.0 mm·min^-1^ for each set of experiments based on the frequency of heavy rainfall in the Wenchuan earthquake area. The experimental design included 2 treatments (the weight proportion of gravel and rainfall intensity) with 3 levels and 3 repetitions of each set for a total of 27 artificial rainfall testing fields.

**Fig 3:**
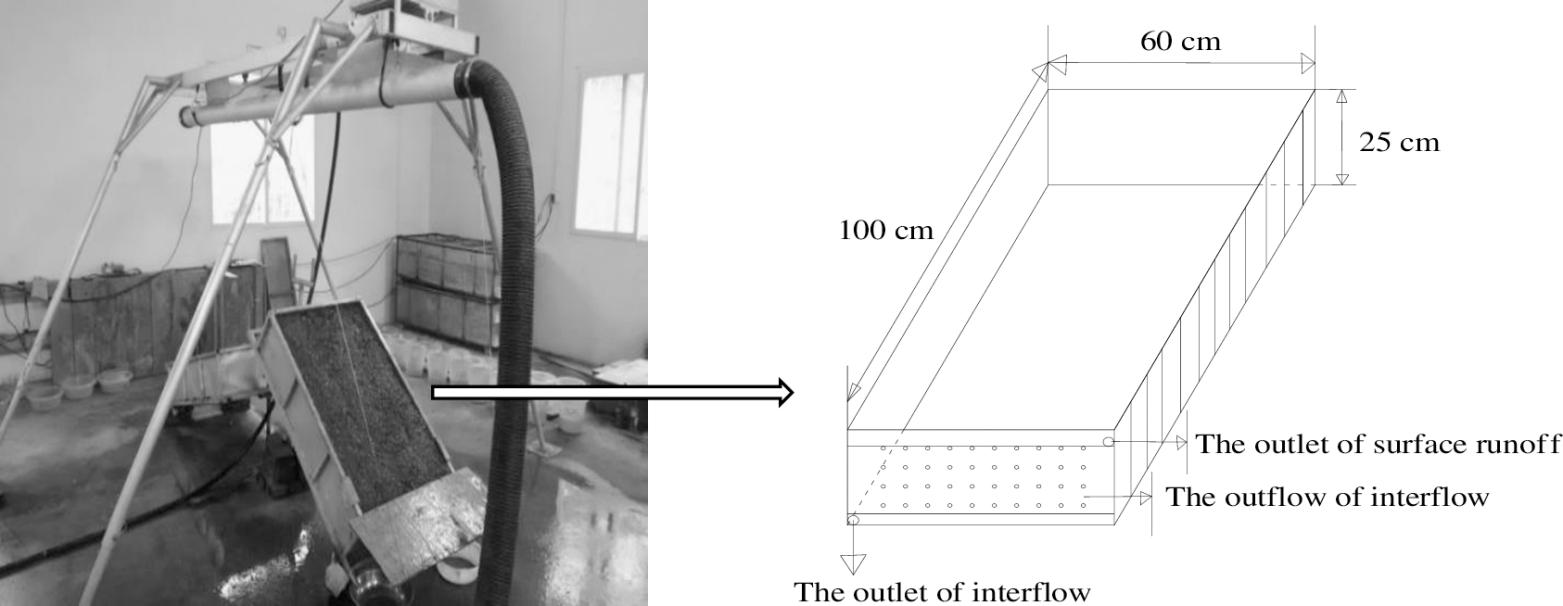
The artificial rainfall simulator used in this study.

The rainfall experiments were conducted in the artificial simulation rainfall hall of Southwest University, Beibei District, Chongqing China. The artificial rainfall simulator was made by the USDA-ARS Soil Erosion Research Laboratory and included the rainfall system, water supply system, and the control system, which was similar to that described by Cerdàerdna. Th7]. The height of stainless steel down-sprayers is 6 m, the effective area of rainfall is 3 m×3 m, and the rainfall intensity can be remote adjusted from 0.2 to 3.2 mm·min^-1^ with final raindrop velocity of more than 85% of natural rainfall. The adjustment time is about 30 s, and adjustment precision is 0.1 mm·min^-1^. The soil rainfall flume was a homemade steel tank with holes punched in the bottom for the interflow measurement (a length of 1.0 meters, a width of 0.6 meters and a depth of 0.25 meters). Then, 2 collection tanks were set up in the upper and middle sections of the flume to collect surface runoff and interflow, respectively. To prevent the soil particles from leaking out from the soil rainfall flume and the water from flowing, the bottom hole zone was plugged by a crude fiber before it was filled with soil (Fig 3). The mixed soil and gravel were filled in the flume with 5 centimeters of compaction each, the actual thickness of the mixture was approximately 24 cm, and the actual dry density of the mixture was approximately1.77g·cm^-3^. The water was divided evenly to make the mixture’s moisture content about 8.25%-12.60% to ensure a good uniformity of the initial mixture’s moisture content. Then, the mixture was permitted to stand for 24 hour was covered by plastic film.

To keep the rainfall uniform and the intensity at the designed experimental indexes, the rainfall intensity was determined for calibration before the experiment. Each experiment was designed to last for 90 min, but some of the experiment lasted longer than 90 min based on testing with a stopwatch. Once the runoff started, the runoff and sediment was collected with an empty plastic container while recording the runoff generation time. When the surface runoff was initiated, the surface runoff and sediment samples were collected in 1 min intervals during the first 10 min, then collected at 3 min intervals for10-40min and finally at 5 min intervals during the 40~90min period.

### 2.4 Measurements and data analyses

Soil infiltration rate (*K*) was measured using the calculating formula (1):

$$K=r\;cos\left(\theta \right)\text{-}\frac{k∙F}{A∙t}$$ (1)

Where *r* was rainfall (cm·min^-1^), *θ* was slope (in °), *t* was time interval (min), *F* was runoff during the time interval *t* (g), *A* was the cross-sectional area of soil rainfall flume (cm^2^), *k* was the converting factor from runoff volume to water volume, *k* = 1 cm^3^·g^-1^.

The soil porosity was measured by the Loop-knife method. Runoff was measured using homemade large measuring cup (L) while the runoff volume was obtained by filtering the sample of water-sediment mixing. The sediment yield rate was calculated by sediment yield during the unit time (g·min^-1^) while the sediment yield include both the suspended sediment and the coarser particles. The sediment samples—collected during the time intervals was dried and then weighted in order to get result in a mass divided by the sampling duration. The cumulative sediment yield (g) would be obtained from the cumulative sum of these weights through time. So the cumulative sediment yield was measured by the product of the total sediment dry weight (g) and the sediment time (min) during the unit time (g). The size of eroded particles came from the suspended sediment and bed load sediment. The runoff velocity was measured by the portable current meter at every 0.5 m during the unit time (m· s^-1^). The regression analysis was conducted to analyze the relationship among the proportion of gravel, runoff generation, sediment yield, and rainfall time. Based on the statistical experiments, all of the data in the tables and figures represent the mean values of each site. The SPSS Statistics 19.0 software was used to conduct the statistical analyses.

## 3. Results

### 3.1 Infiltration characteristics of landslide sediments

**Fig 4:**
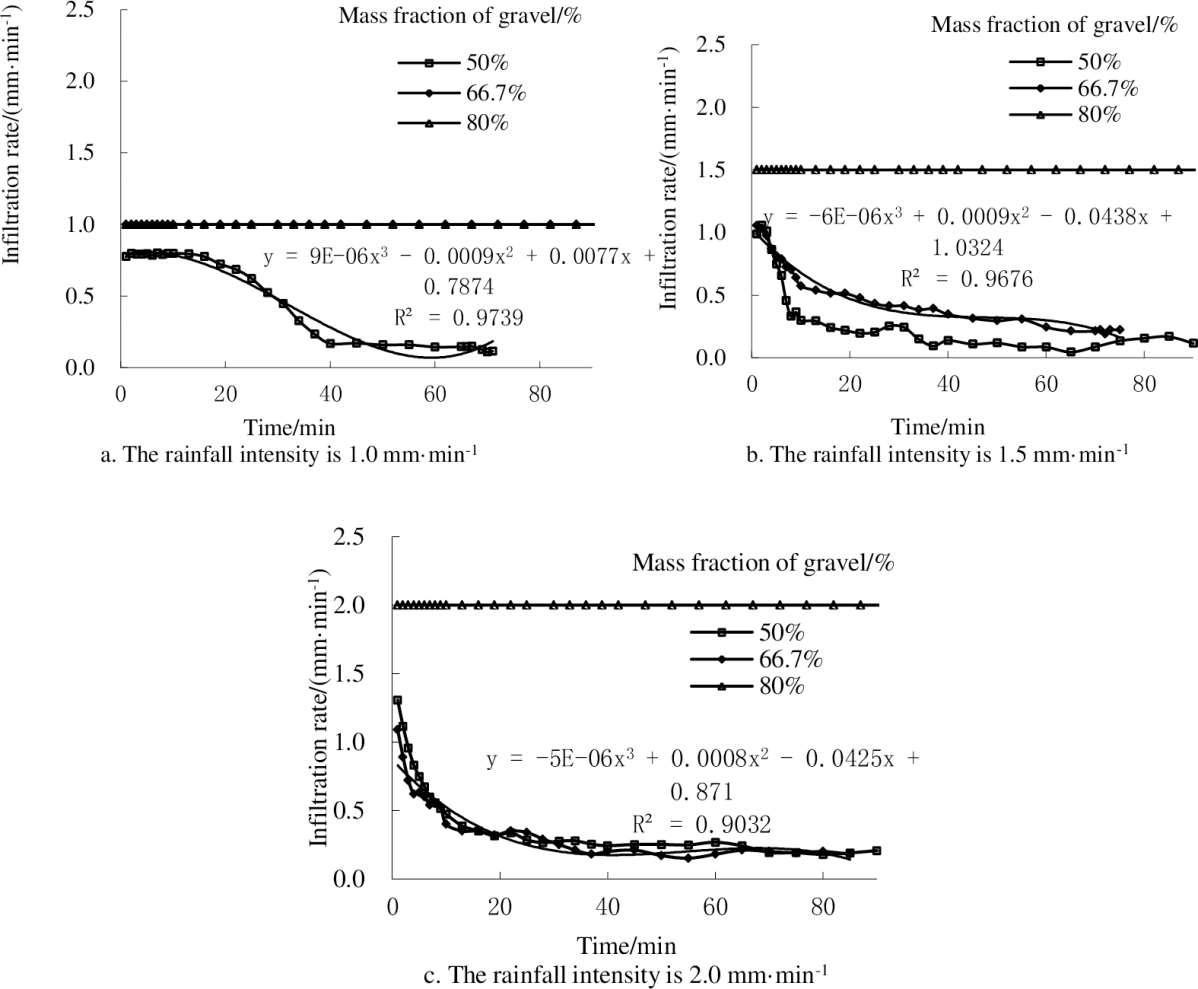
Changes in infiltration rate with rainfall time under different proportions of gravel at different rainfall intensities. Points indicate the average of three replicate experiments.

Fig 4 shows that the weight proportion of the gravel and the main impact on the infiltration capacity in the reconstructed mixtures. The infiltration rate of landslide accumulation was always equal to the rainfall intensity during rainfall processes such as the 80% proportion of gravel under the experimental designs of rainfall intensity and 66.7% proportion of gravel at 1.0 mm·min^-1^ rain intensity. The steady-state infiltration rate in 50% proportion of gravel was always less than that in 66.7 and 80% proportion of gravel when rainfall intensity is 1.0 and 1.5 mm·min^-1^, except that it was slightly larger in 50% proportion of gravel than in 66.7% proportion of gravel when rainfall intensity is 2.0 mm·min^-1^, respectively (Fig 4). The steady-state infiltration rate was in an order of 50<66.7<80% in the experiment.

### 3.2 Runoff characteristics of landslide accumulation

#### 3.2.1The runoff generation time (T) and runoff velocity analysis

**Fig 5:**
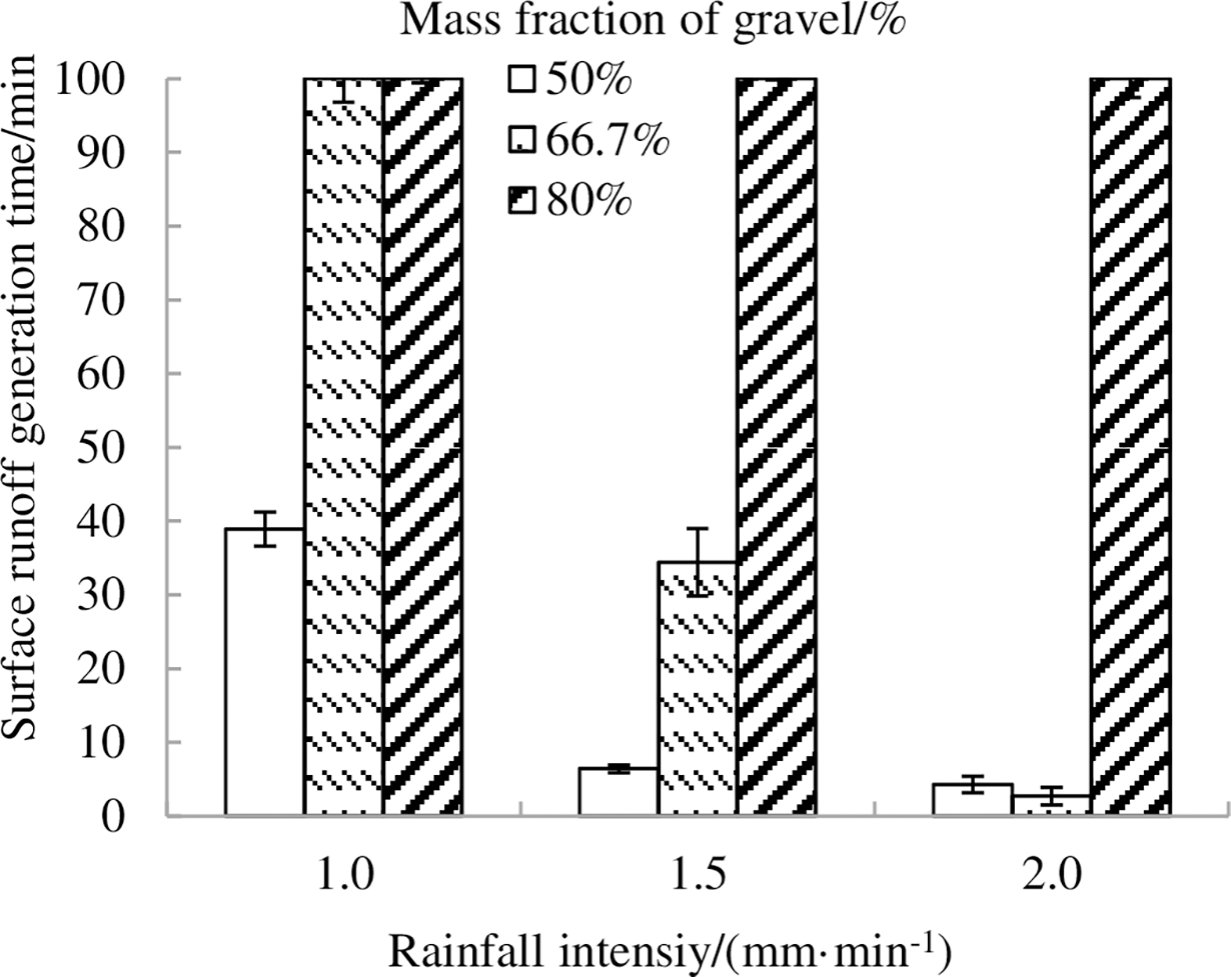
Experimental runoff generation time on landslide sediments for three fractions of gravel with rainfall intensity.

Fig 5 shows that the weight proportion of the gravel had a major impact on the runoff generation time (T) with different rainfall intensities. By observing the whole experiment, stored-full runoff was the main pattern of runoff yield in Landslide deposits. The 50% proportion of gravel was the first to result in rainfall runoff. The change trend of runoff generation time (T) in the 50% proportion of the gravel is similar to that in the 66.7% proportion of gravel. However, no runoff occurred during experiments with 80% proportion of gravel. No runoff occurred for the 66.7% proportion of gravel at a1.0 mm·min^-1^ rainfall intensity.

The runoff generation time (T) in 80% proportion of gravel was longer than those of the 50% and 66.7% proportions of gravel. However, with uneven fill soil, the runoff generation time (T) of the 66.7% proportion of gravel was less than that of 50% proportion of the gravel under the 2.0 mm·min^-1^treatment. In general, the Fig 5 shows two major trends: (1) when intensity increases, runoff generation time decreases (which could arise because saturation is reached more quickly or because excess-saturation runoff is combined with excess-infiltration runoff); (2) when gravel proportion increases, runoff generation time increases (which could mean that more time is necessary to reach saturation likely because of the rise of porosity).We can also see the runoff generation time is different for the gravel fraction which showed that there was a threshold in the gravel fraction on the effect of runoff rate. If the gravel fraction went below the threshold value, the runoff generation changed with the rainfall intensity. When the gravel fraction was more than the threshold value, the rainfall intensity had no effect on the runoff generation under such condition of gravel fraction.

**Fig 6:**
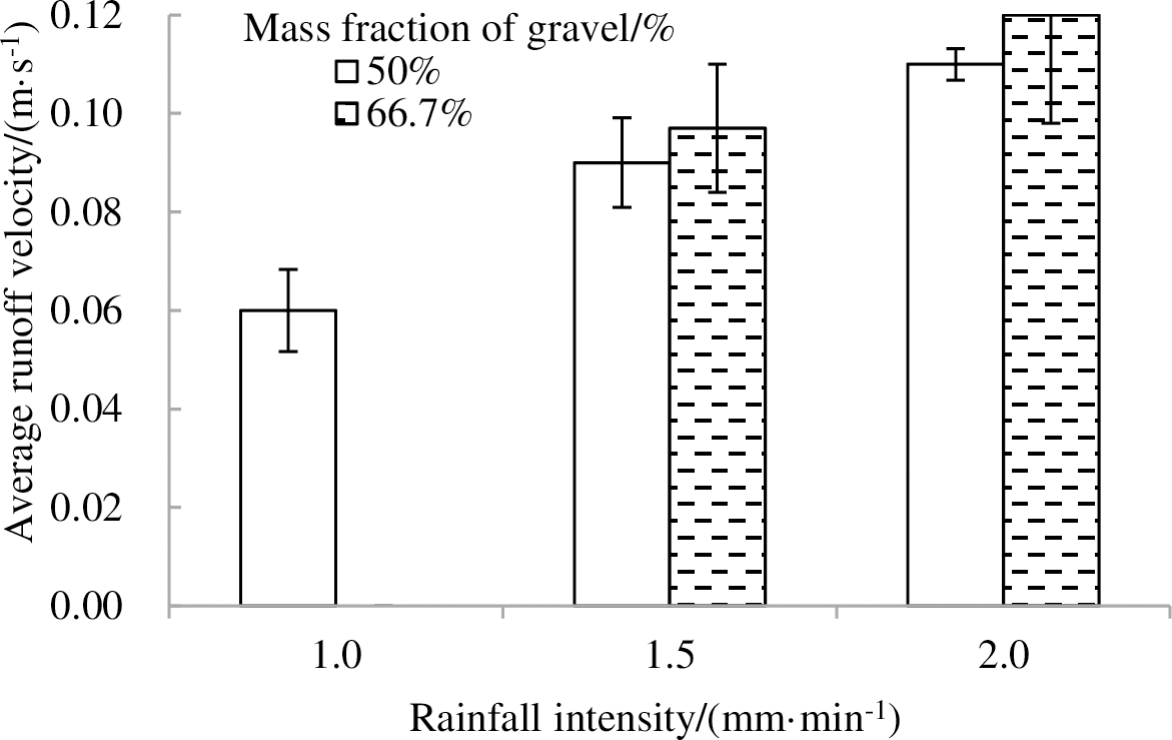
Variations in average runoff velocity on landslide sediments.

Fig 6. shows the variations of average runoff velocity on the landslide accumulations for three fractions of gravel with rainfall intensity. The average runoff velocity of the landslide accumulations increases with an increase in rainfall intensity. With different soil textures in the landslide accumulation, the average runoff velocity in 50% proportion of gravel was always less than that in 66.6% proportion of gravel, while the average runoff velocity was almost equivalent in 66.6% proportion of gravel when rainfall intensities were 1.5 and 2.0 mm·min^-1^. Meanwhile, the average runoff velocity was in an order of 66.6>50>80% in most rainfall intensities (Fig 6).

#### 3.2.2 Variation characteristics of the runoff rate analysis

**Fig 7:**
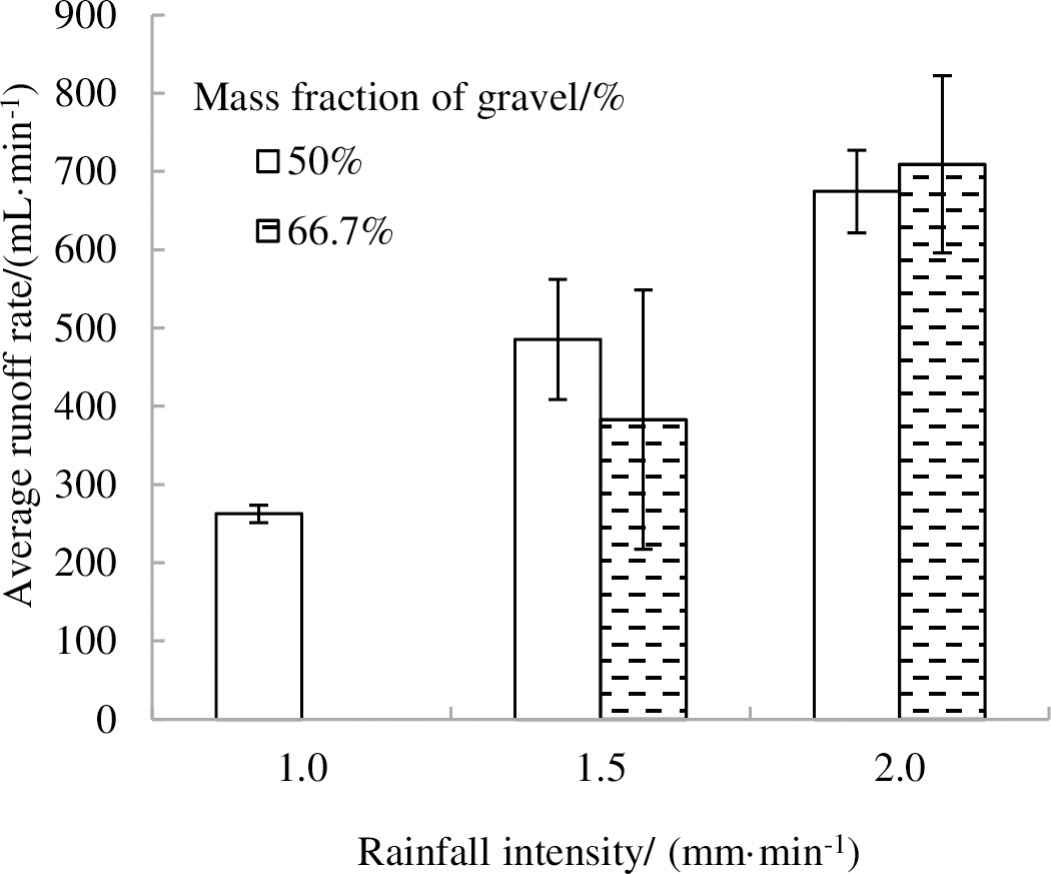
Average runoff rate on landslide sediments for three rainfall intensities with gravel content.

Fig 7 shows the variations of the average runoff rate on landslide accumulations for soil textures with different gravel contents. The average runoff rate increased with increasing rainfall intensity. However, the 50% and 66.6% were similar given the uncertainties at 1.5 mm·min^-1^ and 2.0 mm·min^-1^ runoff intensities.

**Fig 8:**
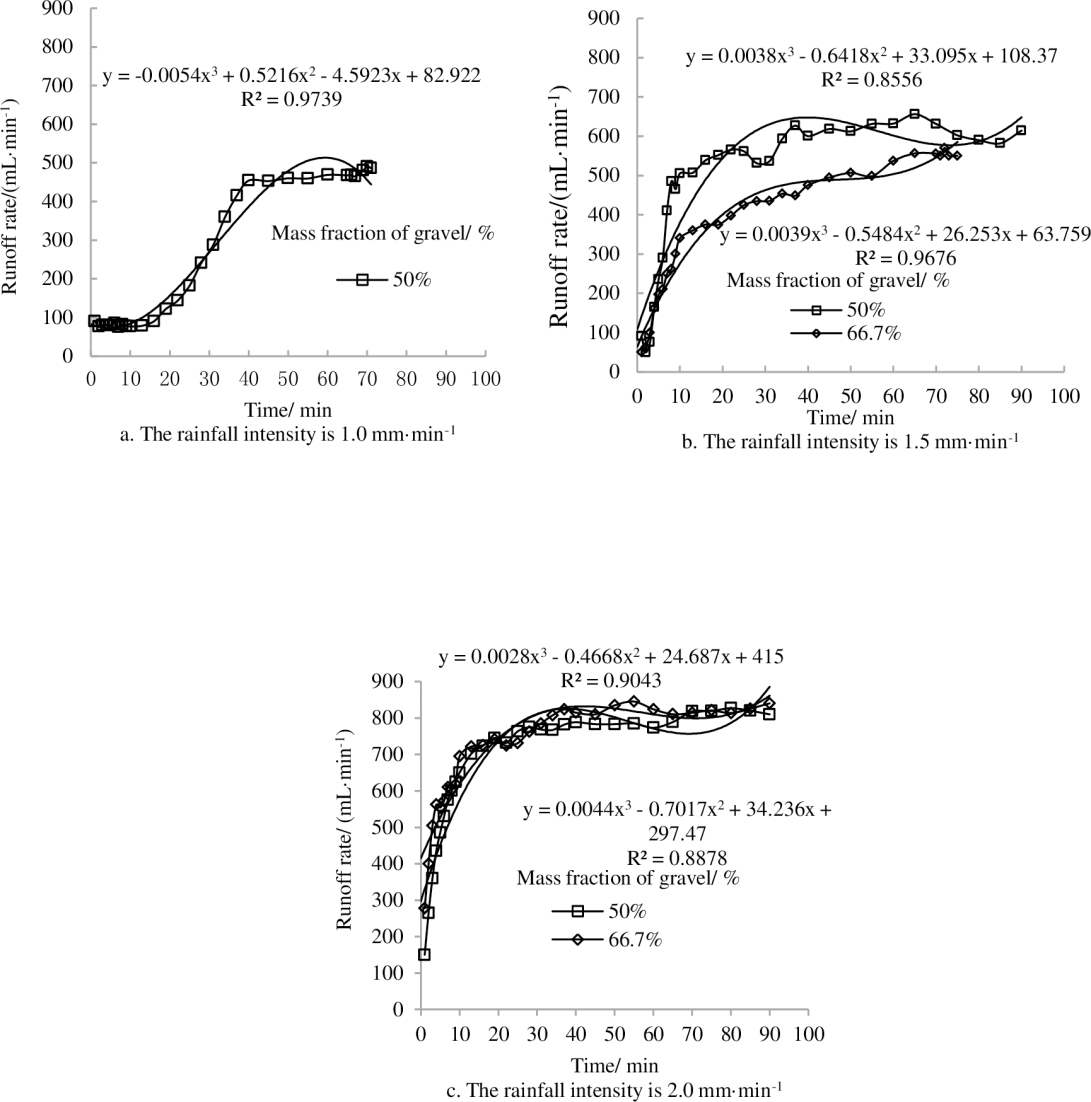
Runoff rate as a function of rainfall time under different proportions of gravel and different rainfall intensities.

In the Fig 8, we can see that there was no runoff occurred during rainfall processes such as the 80% proportion of gravel under the experimental designs of rainfall intensity and the 66.7% proportion of gravel at a1.0 mm·min^-1^ rain intensity. The runoff rate increased gradually at the beginning of rainfall duration on both proportions of gravel and rainfall intensities and then tended to stabilize. The steady-state runoff rate of the 50% proportion of gravel was 80.8 mL·min^-1^ in 15 min, after which the runoff rate grew continually until approximately 40 minutes, then the runoff stabilized (Fig 8A). When the rain intensity was 1.5 mm·min^-1^, the runoff rate in the 50% proportion of gravel was greater than that of the 66.7% proportion of gravel and the steady-state runoff rate of the 50% proportion of gravel, which was significantly greater than that of the 66.7% proportion of gravel. Further, the runoff rate was mainly linked to rainfall time (Fig 8A–8C).

### 3.3 Sediment yield characteristics of landslide accumulation

**Fig 9:**
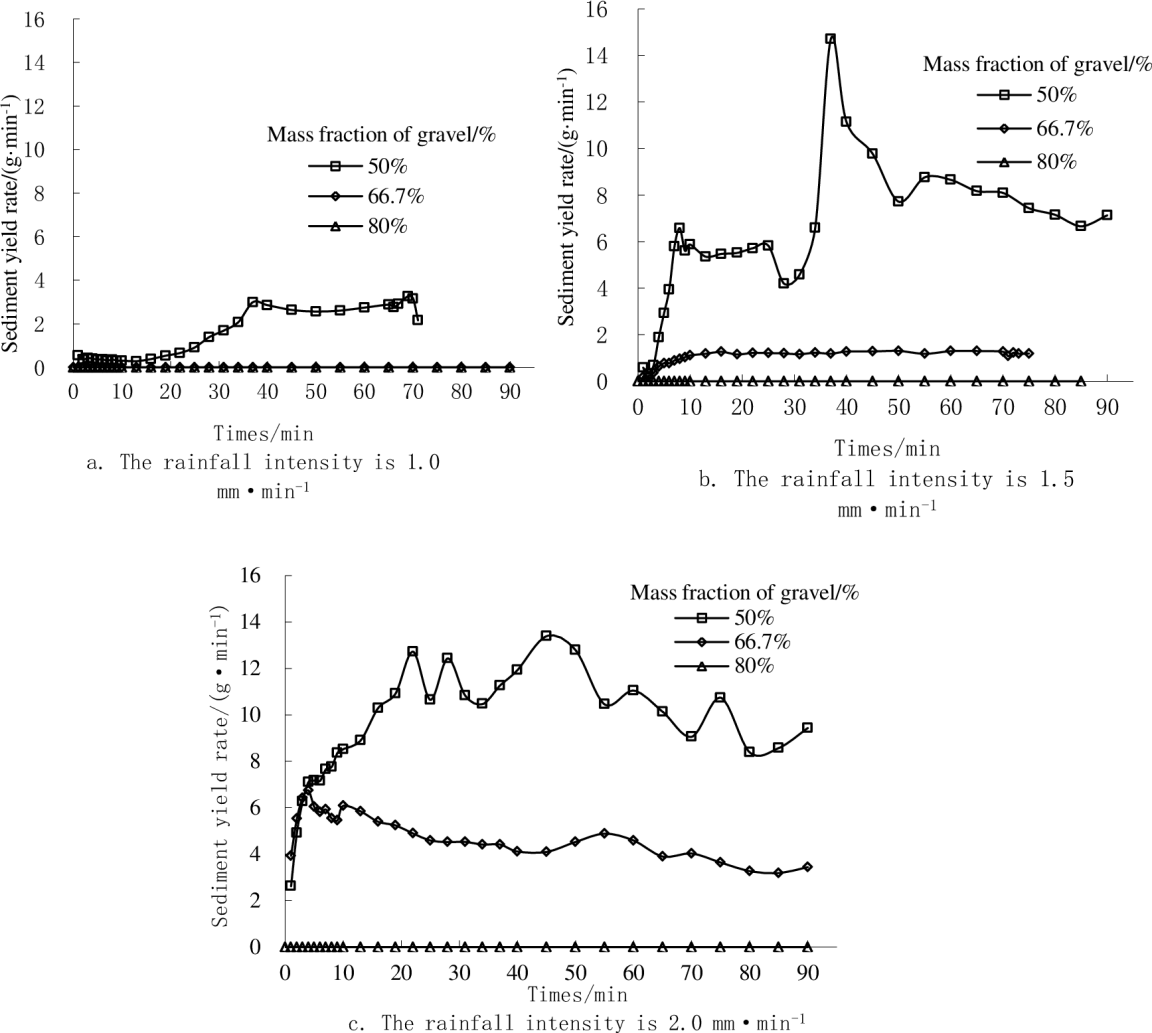
Sediment yield rate as a function of rainfall time under different proportions of gravel and different rainfall intensities.

Fig 9 shows the variations of the sediment yield rate on landslide accumulations for soil textures with different gravel contents. In total, the sediment yield of the 50% proportion of gravel was greater than that of the 66.7% proportion of gravel under the same rainfall intensity. The average sediment rate of the 50% proportion of gravel and 66.7% proportion gravel was 5.48 and 2.84 g·min^-1^, respectively. When the rainfall intensity was 1.0 to 2.0 mm·min^-1^, an increase in the sediment yield occurred in the early stages of rainfall, and then the sediment yield showed a fluctuating growth trend in the middle stage of rainfall, and it finally reached a steady state value when the rainwater washing out ability reached a stead state value. Meanwhile, we can see that the stable sediment yield rate changes of the 50% proportion gravel was in an order of 1.0<1.5<2.0 mm·min^-1^ in rainfall intensities. The increased trend of the average sediment yield rate in rainfall intensities, which ranged from 1.0 to 1.5 mm·min^-1^ was less than that of rainfall intensity from 1.5 to 2.0 mm·min^-1^. Meanwhile, the average sediment yield rate of the 66.7% proportion gravel was in an order of 1.0<1.5<2.0 mm·min^-1^ in rainfall intensities. In total, the average sediment yield rate was in an order of 50>66.6>80% in most experiments.

### 3.4 Consistency between runoff variation and sediment variation

**Fig 10:**
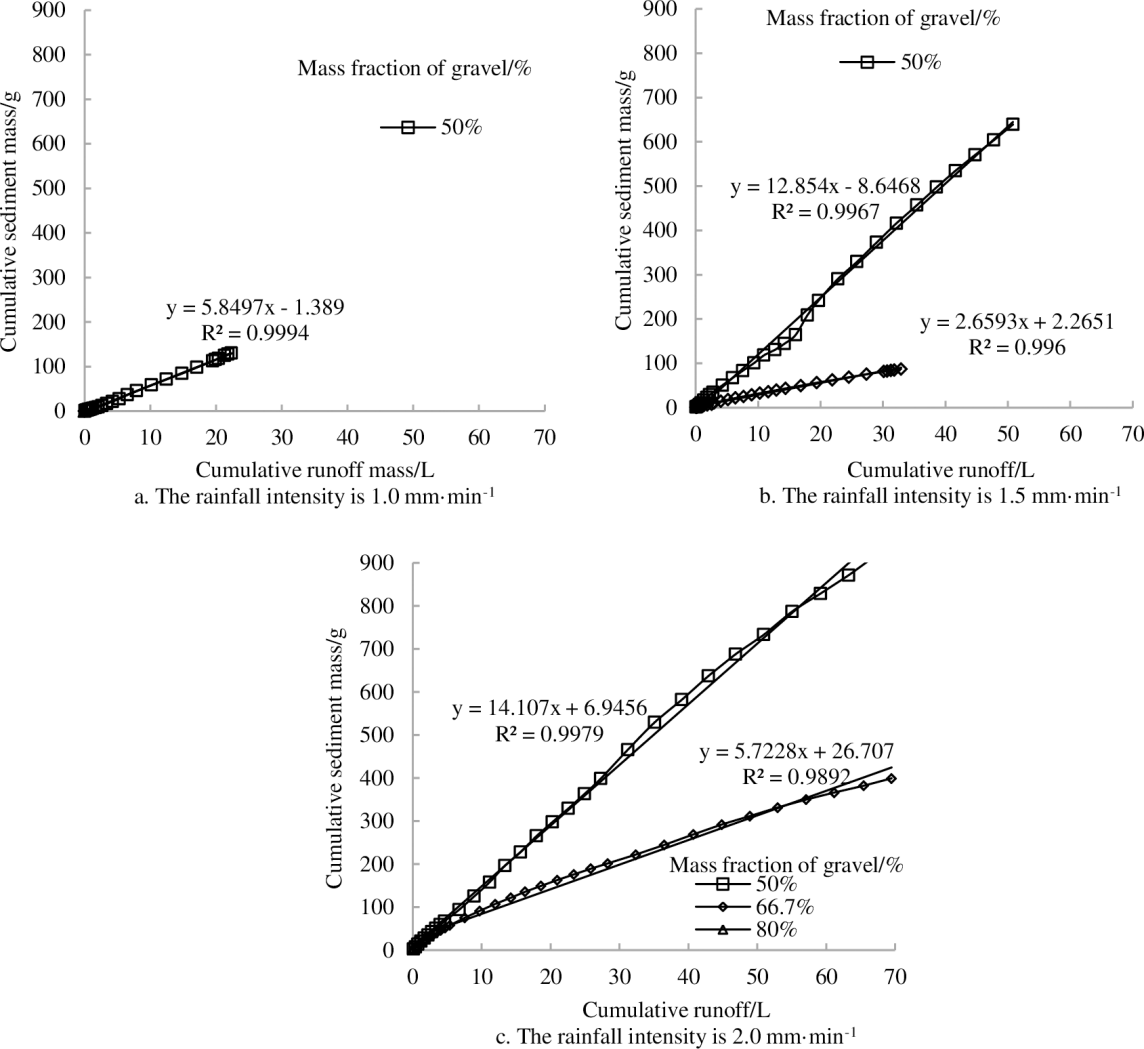
Relationship between cumulative runoff and cumulative sediment mass as a function of rainfall intensity and gravel proportion.

Fig 10 shows the changes in cumulative sediment mass and cumulative runoff on landslide accumulations for soil textures with different gravel contents. Under certain proportions of gravel, the slope of the sediment-runoff curve increased with the increasing of rainfall intensity. Meanwhile, the runoff yield was not synchronous with sediment yield and had a certain hysteresis for the runoff yield. From the figure it can be seen that the relationships between cumulative sediment mass and cumulative runoff is positively correlated which can be described by a linear function (Fig 10):

U=a×Q+b (2)

Where *U* is the cumulative sediment mass, *Q* is the cumulative runoff, *a* and *b* are parameters. All fitting determination coefficients (*R*^*2*^) are larger than 0.9 and reach significance level. This shows that the established models based on multiple simulation rainfall results can be used for predicting the trend of water and soil loss in Wenchuan earthquake area.

## 4. Discussion

Infiltration is one of the most important factors in water and soil loss processes and can directly affect the runoff production and sediment yield. The rainfall intensity and the proportion of gravel had a high correlation degree of infiltration capacity [27–28]. Geng et al. (2009) [29] determined that the rain kinetic energy could promote the formation of soil surface physical crust and reduce the soil infiltration under the condition of rainfall intensity, which was increased to 100 mm·h^-1^. Wang et al. (2014) [30] calculated that the infiltration capacity was reduced when the content of rock fragments was 30%, but it enhanced when the content was less than 30%. In our case, we can see the soil infiltration capability is larger at the beginning of rainfall, the soil infiltration rate is less than the rainfall intensity, and the infiltration ability tends to stabilize gradually. In addition, the infiltration rate is highest at 80% proportion of gravel while gradually decreases under 66.7 and 50% proportion of gravel, respectively. This was because the gravel changed the infiltration mode.

The runoff characteristics of landslide accumulation showed a considerable difference among the three proportions of gravel under rainfall intensity. When the proportion of gravel is increased from 50 to 80%, the runoff rate decreased with the decreasing of rainfall intensity. However, from the Fig 7 it can be seen that for 66.7% proportion of gravels, at 1.0 mm·min^-1^ there is no runoff, at 1.5 mm·min^-1^ there is less runoff than for 50% proportion of gravels and at 2.0 mm·min^-1^ there a similar runoff as for 50% proportion of gravels. The characteristic of the surface runoff is closely related to the soil infiltration ability. Meanwhile, when infiltration rate or runoff rate stabilize, the saturation is reached and surface runoff is generated.

The results herein may be due to the following reasons. First, the soil permeability variation is slight when the proportion of gravel is not changed. The rainfall infiltration capacity increased with the increasing of rainfall intensity [31]. The variation amplitude of the runoff rate is higher when the rainfall amount is lost along with the soil surface. Second, when the rainfall intensity is increased 2.0 mm·min^-1^, the effect associated to gravel proportion on the infiltration capacity becomes negligible in comparison with the difference between infiltration capacity and rainfall intensity. It is also possible that there is no runoff for 80% gravel proportion because the infiltration capacity is too high for the larger soil porosity, only a longer experiment allowed to reach saturation would have led to runoff. So there was a critical point between the 50% and 60% proportions of gravel at the same rainfall intensity and that beyond this point, the rock fragments had no significant effect on the slopes-runoff. And the critical point between 50% and 60% proportions of gravel is possibly due to the non-monotonic relationship between porosity and number of rock fragments in a soil-rock mixture.

The process of the sediment yield with time can be divided into three stages [32]: 1) an increasing phase, where the sediment yield increased with increasing rainfall intensity. There was much more loose surface soil on the landslide deposit slope in the early stage of rainfall, and the sediment yield increased rapidly under the effect of rain wash. 2) A fluctuation phase in which erosion on the landslide deposit slope grew quickly under the rain wash out and the loose surface soil on the landslide deposit slope was generally exposed, which reduced the slope sediment. However, under the effect of geo-potential and rain wash out, a ditch side was formed when the landslide deposit slope, which collapsed periodically so the increased sediment yield showed a fluctuating trend in growth. 3) A steady development phase in which the rainwater washing out ability and sediment yield reach a steady state value.

Ricke-Zapp et al (2007) [33] conducted a similar experiment in the landscape and found that a certain amount of the rock fragments could reduce the sediment yield. Kou-Chan Hung et al. (2007) [34] indicated that the rock fragment had a different effect on hydrologic conditions, depending on the starting conditions. Under wet starting conditions, the rock fragments accelerated the sediment yield, but the sediment yield can also be delayed. In addition, our results indicated that the sediment yield was found to increase at the beginning of rainfall, and then decrease in the 50% proportion of gravel. However, the soil erosion was lower in the 66.7% proportion gravel.

In summary, the change process of sediment yield on the landslide accumulation had the following characteristics: 1) at a given proportion of gravel, the sediment yield increased with the increasing rainfall intensity. This could have occurred because the soil aggregate structure under the low rainfall intensity was so loose that the rain infiltration ability became strong. When the rainfall intensity increased, a crust formed on the slope surface of the landslide accumulation, water-holding capacity decreased, soil moisture gradually became saturated, and the runoff yield and sediment yield increased gradually with the increasing of sediment yield. 2) When the rainfall intensity was a certain value, the sediment yield decreased with the increasing of rock fragments. This may have occurred because of the obvious variation of particle composition in the landslide accumulation. The sediment concentration in the 50% proportion of gravel was significantly higher than those of the 66.7% and 80% proportions of gravel, and in the process of soil filling, the soil porosity was larger with the smaller rock fragments, the loose sand filled in the landslide accumulation under the effect of gravity, the loose sediment concentration was relatively small on the slope, and the bare rock formed a protective layers the lower-accumulation would not be eroded by water. 3) The physical properties of the landslide accumulation were different under the different rock fragments and rainfall intensities at the beginning of rainfalls, and the sediment yield was different under the impact of rain. In addition, the sediment yield appeared to show variability as the rainfall continues. Therefore, it was necessary to study and analyze from the view of hydrodynamics and that the runoff energy was the conveyer for loose sand on the slope surface at the beginning of rainfall. In addition, surface runoff is one of the motivating factors for sediment loss and the procedure of interactions between surface runoff capability and anti-erosion ability when determining the sediment yield on landslide accumulation. The runoff in the whole rainfall movement process can be viewed as the energy producing process of rainwater. Greater rainfall intensity results in stronger washing out ability for sediment detachment [35]. At the end of the rainfall, the runoff gradually stabilized which served mainly to overcome the resistance of the sediment particles on the landslide accumulation. The sediment yield came mainly from denudation and dispersion by runoff. These results were similar to those of the previous studies, which illustrated that the rock fragments were able to reduce and promote erosion over the entire period of rainfall.

With the data obtained from field investigation, the landslide accumulation in Wenchuan earthquake had several distinguishing points. The soil particle size of the landslide accumulation changed i.e. the fine particles in the upper slope, the mid-gravel in the middle slope and the larger size of the stone in the lower slope. The scale of the loose landslide accumulation in Wenchuan earthquake was about 50×10^8^−100×10^8^ m^3^. Under the condition of rainfall and surface runoff, the loose accumulated materials can be transported into the streams, lakes and oceans, resulting in amounts of soil and water loss. Meanwhile, the loose accumulated materials, the sediment source of debris flows, are easily to be mobilized by the sufficient supply of water, destroying the reconstructed infrastructures and endangering the resettled residents. Most investigators believe that rock fragments are one of the key factors in soil erosion and sediment yield [36–37]. Saleh H et al. (2009) [38] concluded that jessour stone terraces had significantly higher sedimentation (64.6 g·m^-2^) and runoff (36.1 L·m^-2^) than did the control stones (natural vegetation, stone terraces and contour ridge). Van Wesemael B et al. (1995) [39] studied smaller rock fragments that were smashed by larger ones, to certain degrees, as found among rock fragments that were removed from the plough layer. Li et al. (2016)[40]found that soil erosion occurred in the following sequence of 50<0<33.3<25%, which agreed with the average sediment yield with which gravels can reduce soil erosion by dissipating energy in the scouring flow rates. In our study, we can see the gravel could increase the water infiltration by increasing the water flow pathways and soil macropores. The change of infiltration is an important factor influencing the process of soil erosion by the change of the surface runoff in landslide accumulation. In addition, the soil containing rock fragments is easy to develop concentrated runoff that results the intensive soil erosion. Various studies [41–42] have shown that the loose landslide accumulation has greatly changed the soil erosion conditions and the sediment source of river which raises the riverbed in the main channel with the greatly increased of the sediment concentration of the river in the earthquake area.

The sediment concentration on the outlet section after the earthquake about 5 times more than the background values before the earthquake [43]. The loose landslide accumulation loss the protective layer of surface erosion can increase the soil erosion rate and the velocity of the slope surface flow. And the exposed bare rock has changed the conditions of infiltration and runoff, which was formed by earthquake. So the soil erosion management of landslide deposits should be relevant in different proportions of rock fragment that meets the basic and long-term protective of local people. For the high proportion of rock fragments, the priority of prevention and control measure should focus on the engineering measures, including retaining wall, intercept grid and so on. Further, it would take some effective auxiliary measures to improve the engineering measures, such as slope surface protection, slope drainage, vegetation restoration and surface consolidation. For the low proportion of rock fragment, it may be more useful to combine biological measures with engineering measures and biological measures.

## 5. Conclusions

This research on soil erosion related to landslide deposits provides new observations which could help guide soil and water loss prediction models for the Wenchuan Earthquake area. We studied the sediment yield and runoff yield under different proportions of gravel content and rainfall intensities using a device to simulate rainfall.

In the experiments, it was found that gravel fraction exhibited important effects on infiltration rate, runoff rate and sediment yield during the rainfall process in the landslide accumulation. For mild gravel fraction (50%), it could improve infiltration capacity but reduce runoff and sediment generation. While for the heavy gravel fraction (80%), there is no runoff and sediment generation during the rainfall process on the landslide accumulation. In addition, rock fragment proportion appeared to have a greater effect on increasing infiltration capacity than runoff generation. Moreover, regarding the response of the rock fragment proportion to the landslide accumulation, both the average runoff rate and sediment yield rate had the following ranks: 50>66.6>80%. Finally, the relationships between cumulative sediment mass and cumulative runoff on landslide accumulation with three different proportions of gravel can be described by a linear function.

## Supporting information

S1 Table Sample weight of different soil-rock ratio. (PDF)

S1 Fig Samples of different particle diameter. (PDF)

S2 Fig Distribution of landslide accumulation body in Wenchuan earthquake area. (PDF)

S3 Fig Elevation of Wenchuan earthquake area. (PDF)

S4 Fig Scene photo of landslide accumulation body in Wenchuan earthquake area. (PDF)
