# Supplementary material for: Water and soil loss from landslide deposits as a function of gravel content in the Wenchuan earthquake area, China, revealed by artificial rainfall simulations
Source: PLoS One. 2018 May 3;13(5):e0196657. doi: 10.1371/journal.pone.0196657 (PMC5933758; doi:10.1371/journal.pone.0196657)
Supplement: S1 Table — (PDF) [file pone.0196657.s001.pdf]

S1 Table. Sample weight of different soil-rock ratio

Particle diameters	Proportion of gravel			Air-dry moisture content
	1:1	1:2	1:4	
Soil (<2mm)	129.50kg	86.33kg	51.80kg	1.568%
Fine gravel (2~10mm)	63.97kg	85.29kg	102.35kg	0.341%
Middle greave (10~60mm)	63.85kg	85.14kg	102.17kg	0.164%
