# Supplementary figures and images for: Water and soil loss from landslide deposits as a function of gravel content in the Wenchuan earthquake area, China, revealed by artificial rainfall simulations
Source: PLoS One. 2018 May 3;13(5):e0196657. doi: 10.1371/journal.pone.0196657 (PMC5933758; doi:10.1371/journal.pone.0196657)

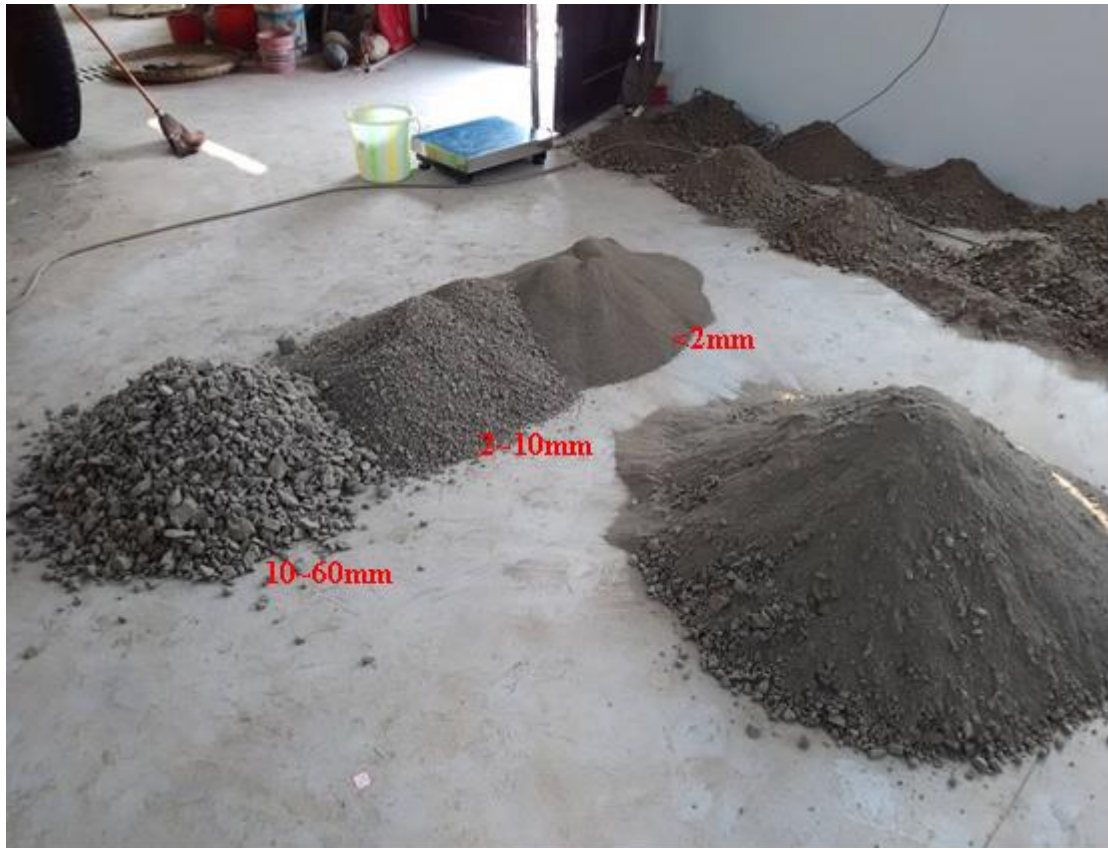

S1 Figure. samples of different particle diameter

Supplement: S1 Fig — (PDF) [file pone.0196657.s002.pdf]

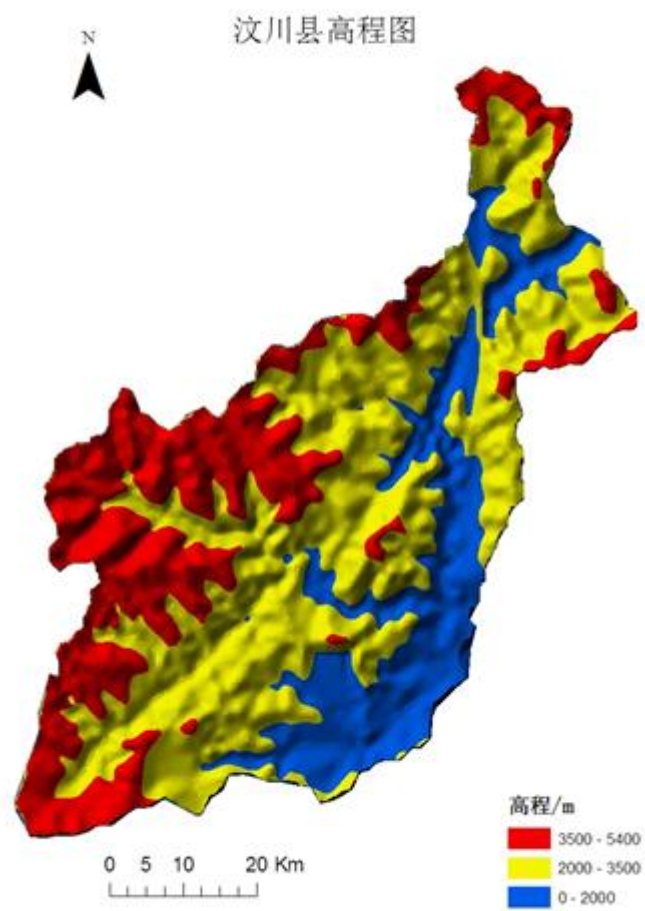

**S3 Figure. Elevation of Wenchuan county**

Supplement: S3 Fig — (PDF) [file pone.0196657.s004.pdf]
