# Supplementary material for: Water and soil loss from landslide deposits as a function of gravel content in the Wenchuan earthquake area, China, revealed by artificial rainfall simulations
Source: PLoS One. 2018 May 3;13(5):e0196657. doi: 10.1371/journal.pone.0196657 (PMC5933758; doi:10.1371/journal.pone.0196657)
Supplement: S2 Fig — (PDF) [file pone.0196657.s003.pdf]

This remote sensing image data of Wenchuan earthquake was collected from the GoogleEarth (spatial resolution about 30m×30m) at different periods after the earthquake for 6 years (2008 to 2014).

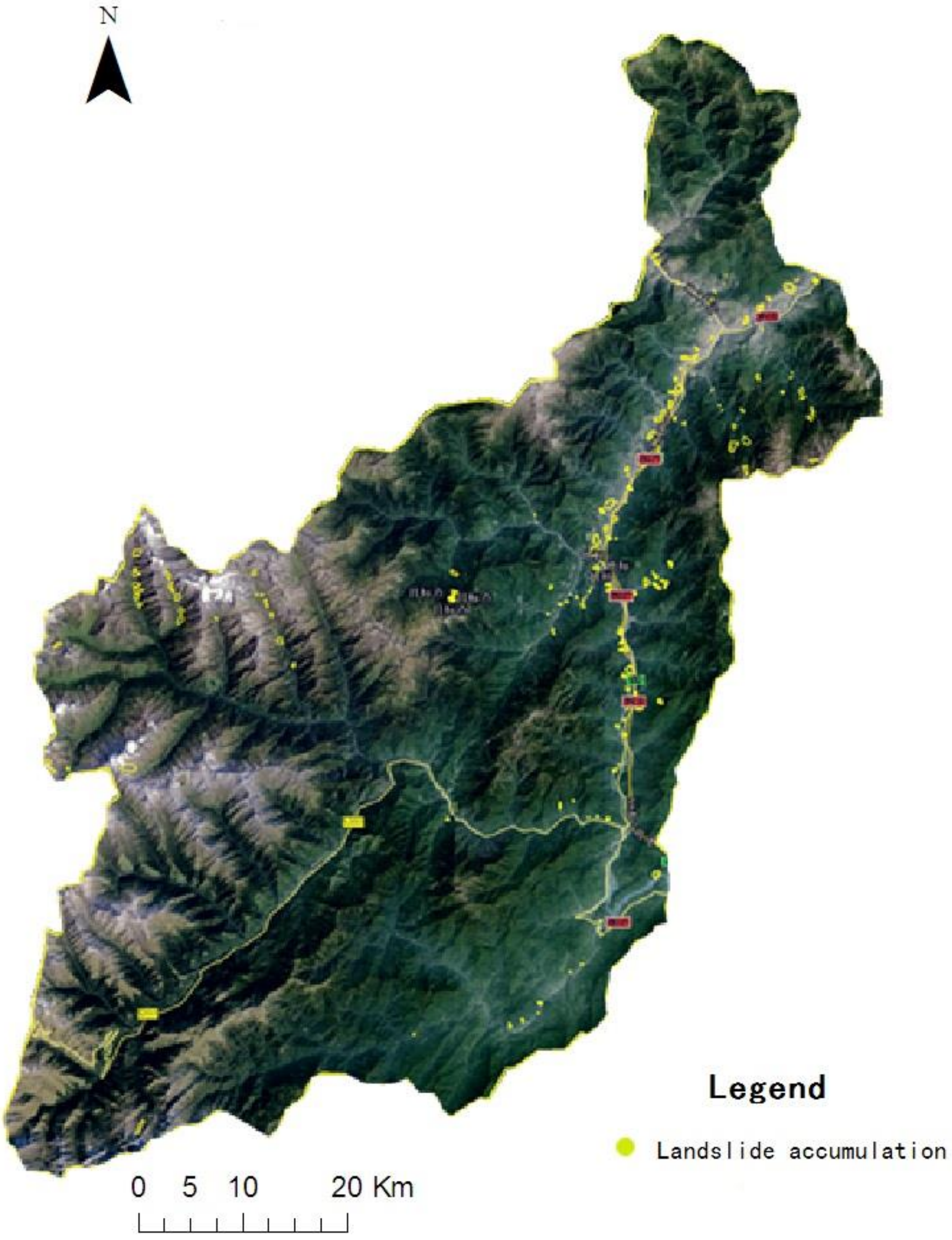

This remote sensing image data of Wenchuan earthquake was collected from the GoogleEarth (spatial resolution about 30m×30m) at different periods after the earthquake for 6 years (2008 to 2014).
