# Supplementary material for: Water and soil loss from landslide deposits as a function of gravel content in the Wenchuan earthquake area, China, revealed by artificial rainfall simulations
Source: PLoS One. 2018 May 3;13(5):e0196657. doi: 10.1371/journal.pone.0196657 (PMC5933758; doi:10.1371/journal.pone.0196657)
Supplement: S4 Fig — (PDF) [file pone.0196657.s005.pdf]

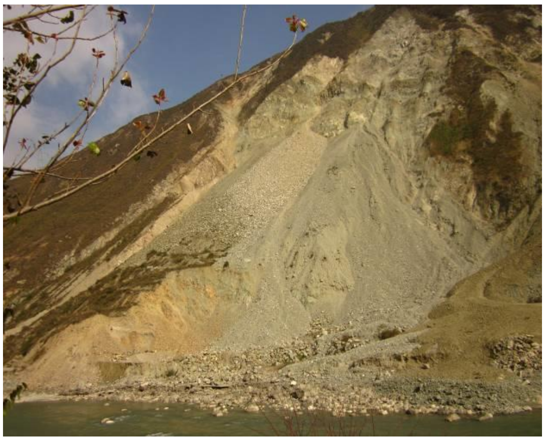

November in 2014

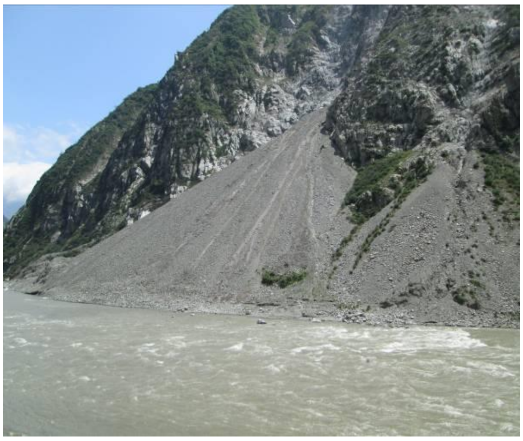

May in 2015

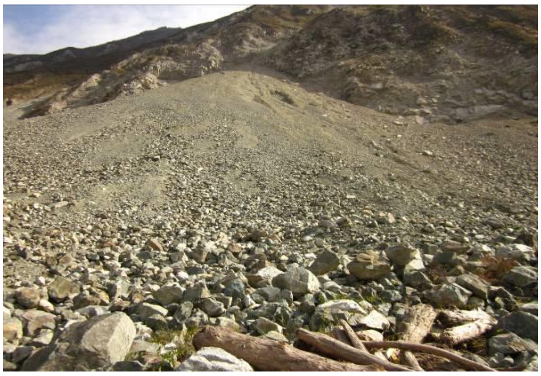

November in 2014

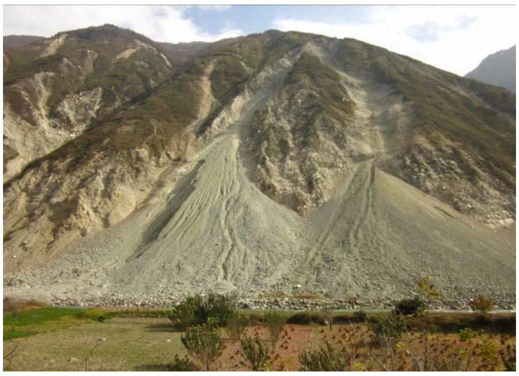

May in 2015

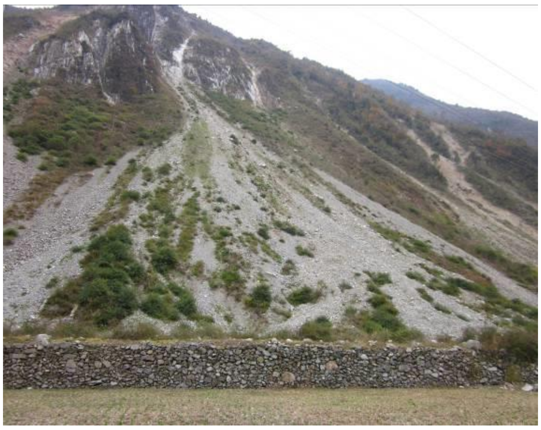

November in 2014

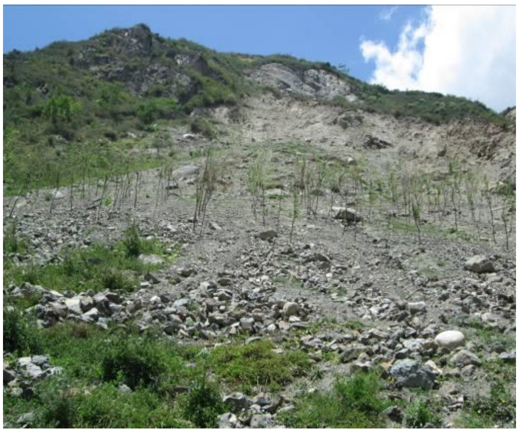

May in 2015

S4 Figure. Scene photo of landslide accumulation body

These photographs were taken from the Mingjiang river (102°51′-103°44′E，30°45′-31°43′N), Chengdu Plain, Sichuan Province, China. The study area is an important ecological barrier of the upper Yangtze River and the Wenchuan earthquake area, which is located in the upriver and headstream of the Mingjiang River, Fujiang River and Jianglingjiang River which provides significant ecosystem services for water conservation or soil and water conservation.
